# Medicinally Privileged Natural Chalcones: Abundance, Mechanisms of Action, and Clinical Trials

**DOI:** 10.3390/ijms25179623

**Published:** 2024-09-05

**Authors:** Sophia M. Villa, Justin Heckman, Debasish Bandyopadhyay

**Affiliations:** 1 Department of Immunology, Harvard Medical School, 77 Avenue Louis Pasteur, NRB 1030, Boston, MA 02115, USA; sophia_villa@hms.harvard.edu; 2 School of Medicine (SOM), University of Texas Rio Grande Valley, Edinburg, TX 78539, USA; justin.heckman01@utrgv.edu; 3 School of Integrative Biological and Chemical Sciences (SIBCS), University of Texas Rio Grande Valley, Edinburg, TX 78539, USA; 4 School of Earth, Environmental, and Marine Sciences (SEEMS), University of Texas Rio Grande Valley, Edinburg, TX 78539, USA

**Keywords:** chalcones, antidiabetic, antiviral, neuroprotective, cardioprotective, antimalarial, antibacterial, anti-inflammatory, antioxidant, clinical trials

## Abstract

Chalcones have been utilized for centuries as foods and medicines across various cultures and traditions worldwide. This paper concisely overviews their biosynthesis as specialized metabolites in plants and their significance, potential, efficacy, and possibility as future medicines. This is followed by a more in-depth exploration of naturally occurring chalcones and their corresponding mechanisms of action in human bodies. Based on their mechanisms of action, chalcones exhibit many pharmacological properties, including antioxidant, anti-inflammatory, anticancer, antimalarial, antiviral, and antibacterial properties. Novel naturally occurring chalcones are also recognized as potential antidiabetic drugs, and their effect on the GLUT-4 transporter is investigated. In addition, they are examined for their anti-inflammatory effects, focusing on chalcones used for future pharmaceutical utilization. Chalcones also bind to specific receptors and toxins that prevent bacterial and viral infections. Chalcones exhibit physiological protective effects on the biological degradation of different systems, including demyelinating neurodegenerative diseases and preventing hypertension or hyperlipidemia. Chalcones that are/were in clinical trials have been included as a separate section. By revealing the many biological roles of chalcones and their impact on medicine, this paper underlines the significance of naturally occurring chalcones and their extension to patient care, providing the audience with an index of topic-relevant information.

## 1. Introduction

**Figure 1:**
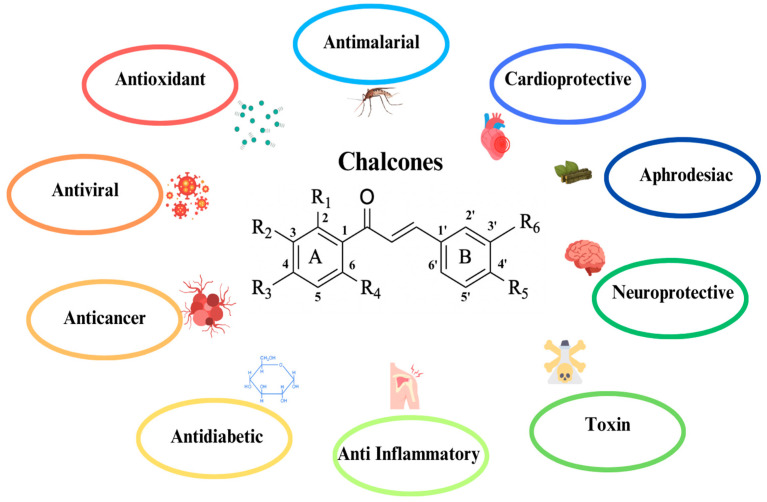
The general structure (carbon skeleton) of chalcones and their biological functions in humans.

Chalcones, also known as chalconoids, represent a class of biologically active and medicinally significant organic molecules [1]. Their carbon skeleton (Figure 1) follows the general formula C6H5C(O)CH=CHC6H5C6H5COCH=CHC6H5, constituted by α, β-unsaturated ketones [2]. A diverse array of compounds falls under the category of chalconoids, primarily as secondary metabolites within plants. Chalcones are prevalent in botanicals traditionally utilized for medicinal or dietary purposes [3]. The general structure and some functions of chalcones are summarized in Figure 1.

There are several categories of chalconoids, including simple or classical chalcones as well as hybrid chalcones, which are synthetically modified or synthesized and exhibit the core structure shown in Figure 1. They can then be further classified into bichalcones, containing two chalcone moieties in one structure, or dihydrochalcones, with a reduced α,β unsaturated double bond [2,3]. When searching for how many chalcones are known, there is an overwhelming number of results, with at least 92,000 chalcones (including chalcone derivatives) being recorded and more than 1000 reporting biological activity [1]. It is important to note that while many chalcones have been synthetically modified to enhance their biological activity, naturally occurring chalcones also exhibit these properties. Given their unique structural moiety, it is not unlikely that naturally occurring chalcones can serve various purposes, including acting as antioxidants, anticancer agents, antidiabetics, antivirals, anti-inflammatories, antimalarials, neuroprotective compounds, and cardiovascular protectants [4]. Because of their many utilities, these medicinally privileged molecules must be investigated further. This review was prompted by the scarcity of information available about purely natural chalcones. There are many literature reviews on synthetic chalcones Although many synthetic chalcones have been used as scaffolds in the pharmaceutical industry, natural chalcones represent a significant area of interest. Thus, as natural compounds possessing a wide range of biological activity, they merit such a compiled review of the available literature, including structures when readily available and verified. It is important to note that all chalcones referred to in this text are naturally occurring and not synthetically modified; the terminology chalcone and natural chalcone are used interchangeably. With this resource, future researchers will be able to locate biologically active, naturally occurring chalcones.

## 2. Biosynthesis of Natural Chalcones

**Scheme 1:**
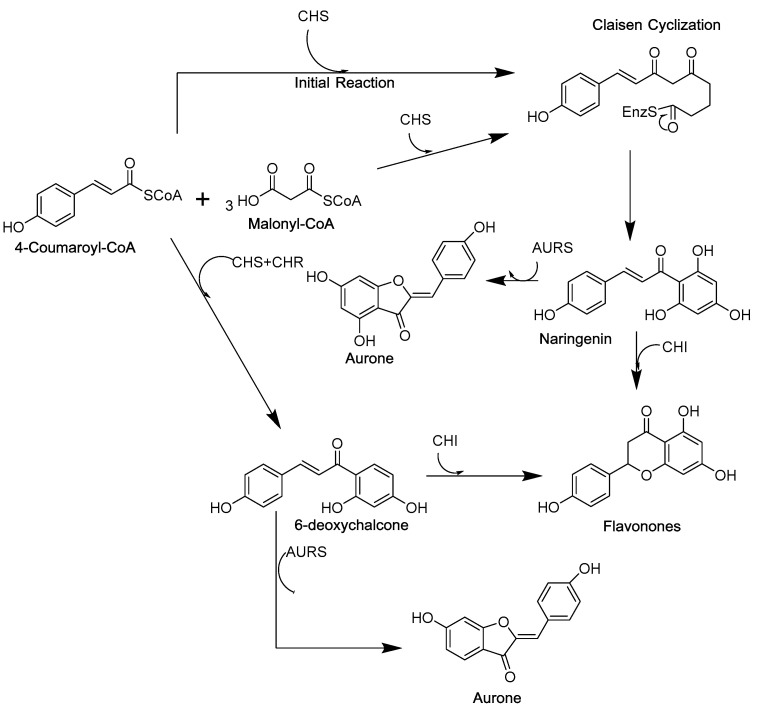
The biosynthetic pathway of chalconoid synthesis (CHS—chalcone synthase, CHI—chalcone isomerase, CHR—Chalcone Reductase, AURS—aurone synthase) [6,8,9].

As secondary metabolites of plants, natural chalcones are not directly involved in plants’ developmental or reproductive functions; instead, they direct interactions with the environment and benefit the organism by increasing their prolific ability [3]. This means that they allow for greater reproduction and environmental flexibility for the plant producing them. For example, if a plant is physiologically stressed by too much exposure to UV radiation or under attack by bacterial, viral, or fungal infections, it will produce secondary metabolites to protect against these stressors. As important secondary metabolites, natural chalcones are synthesized in higher plants by the enzyme chalcone synthase (CHS), a type III polyketide synthase [5]. Type III polyketide synthases are commonly found in higher plants but have been observed in lower divisions of phyla [6]. Any similar enzymes are termed chalcone synthase-like enzymes. Cys160, Phe215, His303, and Asn336 are the active sites consistent across all CHS and CHS-like enzymes [7]. Chalcone synthase (CHS) produces these chalconoid molecules through the flavonoid/isoflavonoid biosynthetic pathway [8]. Noel et al. revealed the mechanism (Scheme 1) by crystalizing CHS, which subsequently provided structural information about CHS and its enzymatic activities [9].

The gene responsible for CHS’s expression (CHS-1) is activated when the plant is under great stress and produces several bioactive protectants against attacking microbial organisms, overexposure to UV light, or injury. Stress ultimately leads to great expression of CHS and to high accumulation of these flavanones, aurones, chalcones, and other secondary metabolites in healthy plant tissues to resist further internal or environmental stressors [9]. The two starting agents, 4-coumaroyl-CoA and malonyl-CoA, are transferred by CHS to Cys164 and are the first step of the mechanism [10]. Three malonyl-CoA thioesters form an intermediate via the polyketide reaction; a Claisen cyclization can then occur, generating the naringenin chalcone [9]. Further action by other enzymes, such as chalcone isomerase (CHI), produces flavanones, while action by plant catechol oxidase, aurone synthase (AURS), leads to the formation of aurone compounds [10]. 

## 3. Medicinal Activity of the Natural Chalcones

Natural chalcones are not just useful for protecting plants; their implementation as privileged scaffolds in medicinal chemistry shows that their natural therapeutic action can be amplified and utilized for in vitro action in the human body. Chalcones are utilized as antioxidants, anticancer, antidiabetic, antiviral, antimalarial, and immunosuppressive agents and have many other pharmacological uses [11]. Besides these general activities, chalcones have even been observed to have aphrodisiac-like effects [12] as well as acting as antimitotic agents and cytotoxins, as seen in *Fissistigma lanuginosum* [13]. These activities may be because of their small structure and Michael acceptor features, which make them readily bind with other biological molecules [14]. Their ability to interact with other molecules and enzymes allows chalcones to intervene and react with several important cellular mechanisms. Chalcones can be considered Michael receptors due to the presence of an α,β-unsaturated carbonyl group in their structure. The Michael receptor molecules are electrophilic due to the presence of carbonyl functionality. These ketones can combine with nucleophilic residues on the protein to form protein-ligand complexes and activate or inhibit the regular protein pathway, which plays an important physiological role. These Michael receptors can regulate the Keap1-Nrf2-ARE and the NF-κB pathways and are considered privileged anti-inflammatory, anticancer, and antioxidative stress agents. This structural feature allows them to readily undergo Michael addition reactions with nucleophiles, molecules, or ions that donate a pair of electrons to form a chemical bond with an electrophile [14].

### 3.1. Chalcones as Michael Acceptors

In biological systems, chalcones can interact with various nucleophiles in the body, such as thiols (-SH) from cysteine residues in proteins or glutathione, an essential intracellular antioxidant [15]. Firstly, in terms of metabolism, chalcones undergo metabolic reactions involving conjugation with nucleophiles such as glutathione, resulting in more water-soluble metabolites readily excreted from the body [16]. This process can directly impact the duration of their action and overall bioavailability. Secondly, chalcones can form covalent adducts with nucleophilic residues on proteins, affecting their bodily distribution and interaction with cellular targets and metabolic enzymes [15]. Depending on the extent of conjugation and the properties of resulting added functional groups or proteins, this can alter the ability of chalcones to penetrate cell membranes or cross biological barriers like the blood–brain barrier [16]. Furthermore, while the Michael acceptor property of chalcones is advantageous for drug design, allowing for targeted biological pathway interactions, it also poses a risk of toxicity [17]. Reactive intermediates formed during such interactions may interact indiscriminately with essential biomolecules, potentially leading to toxic effects [18].

### 3.2. Classification of Natural Chalcones and Their Mechanisms of Action

Classifying chalcones based solely on one type of bioactivity is impossible. Many chalcones exhibit more than one functionality; for example, because of their structure, virtually all chalcones have some form of antioxidant activity. Antioxidant chalcones are also frequently anticancer agents; however, this is not true of all antioxidant chalcones. Thus, a better system is needed to classify and define chalcones and their natural bioactivities. This paper will classify them based on their most prominent activity and utilization as reported in the literature. For example, if a chalcone is a potent antioxidant, it will be presented as such. Sources of the chalcones in their corresponding plants and mechanisms of action will be discussed. This methodology will allow a focus on specific chalcones and utilization, rather than simply listing a magnitude of compounds.

## 4. Chalcones as Antioxidants

### 4.1. Food Chalcones as Antioxidants

Antioxidants are important in the human body because of their crucial role in cellular protection from damage caused by radicals and oxidative stress [19]. Free radicals are unstable molecules that can damage cells and contribute to aging and vulnerability to disease [19]. High concentrations of free radicals can lead to cancer, cardiovascular disease, and even neurodegenerative disorders [20]. Antioxidants scavenge and neutralize these molecules by donating electrons, stabilizing them, and preventing further cellular action [21]. By reducing oxidative stress, antioxidants act as a preventative measure against the negative effects of radical oxygen species [20]. Chalcones exhibit promising antioxidant properties due to their chemical structure and reactivity; their electron-rich phenolic structure makes chalcones ideal antioxidant molecules [22]. Additionally, chalcones have been found to modulate antioxidant enzyme activity and gene expression, further enhancing their protective effects against oxidative damage [22]. Their ability to target multiple pathways involved in oxidative stress makes chalcones valuable candidates for therapeutic interventions to promote health and prevent disease [23]. Furthermore, the abundance of chalcones in dietary sources such as fruits, vegetables, and medicinal plants highlights their potential as natural antioxidants for maintaining overall well-being [24].

**Figure 2:**
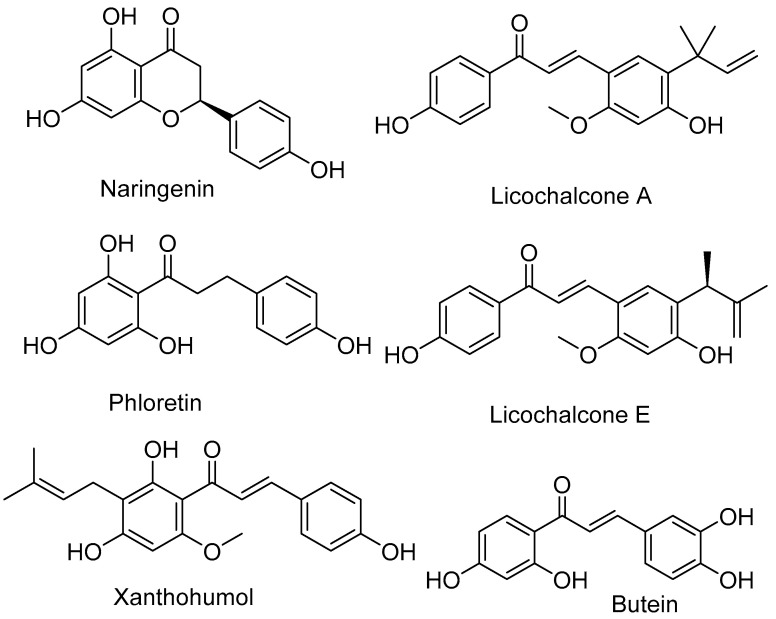
Structures of representative antioxidant chalcones in edible plants and food products.

Antioxidant chalcones exert their effects through various cellular targets and mechanisms. They enhance enzymatic antioxidant defenses such as superoxide dismutase, catalase, and glutathione peroxidase while activating the Nrf2-ARE pathway to upregulate antioxidant and detoxification genes [25]. Additionally, chalcones may inhibit reactive oxygen species (ROS) production by interfering with enzymes like NADPH oxidases and xanthine oxidases, and they possess metal-chelating properties to prevent metal-catalyzed ROS generation [25]. Furthermore, their anti-inflammatory activity, modulation of mitochondrial function, and direct scavenging of ROS contribute to cellular redox balance [26]. Foods rich in antioxidant chalcones offer a natural source of compounds known for their health-promoting properties [27]. Previous studies have noted that some naturally occurring chalcones have even contributed to reducing obesity via inhibiting adipocyte generation, decreasing cholesterol through NPCL1 inhibition, decreasing enzymatic lipase activity, and decreasing lipid accumulation in the adipose layer [28]. These effects are made possible through the contents of citrus fruits like oranges, lemons, and grapefruits. These contain naringenin chalcone, contributing to their antioxidant activity. Similarly, apples provide phloretin, while tomatoes are a good source of naringenin and phloretin. Soybeans and almonds contain isoflavonoids and naringenin, respectively, enhancing their antioxidant profile. Moreover, green tea, red wine, and hops (*Humulus lupulus*) used in beer production are documented sources of xanthohumol, a potent chalcone with antioxidant properties [27]. Butein (Figure 2) is another chalcone isolated from the bark of *Rhus verniciflua* (*Toxicodendron vernicifluum*), while licorice (*Glycyrrhiza glabra*) presents licochalcone A and licochalcone E (Figure 2), further diversifying the array of antioxidant chalcones found in foods [29]. Antioxidant-containing foods are sought after and may be responsible for the health benefits associated with plant-rich diets. These corresponding chalconoids are shown in the structures below.

### 4.2. Further Roles as Antioxidants

Being known for their antioxidant properties is, perhaps, the simplest characteristic of chalcones. Many compounds are strong antioxidants; however, their additional properties are what make chalcones so desired. Having strong antioxidant activity, it ventures into the realm of protection against many chronic diseases. Oxidative stress causes severe damage to nucleic acids, lipids, and proteins, including important enzymes [30]. This destruction is responsible for cancer, arthritis, cardiovascular disease, and neurodegenerative diseases. Various chalcones are responsible for treating and investigating these diseases and will be discussed in the following paragraphs.

## 5. Chalcones with Anticancer Properties

**Figure 3:**
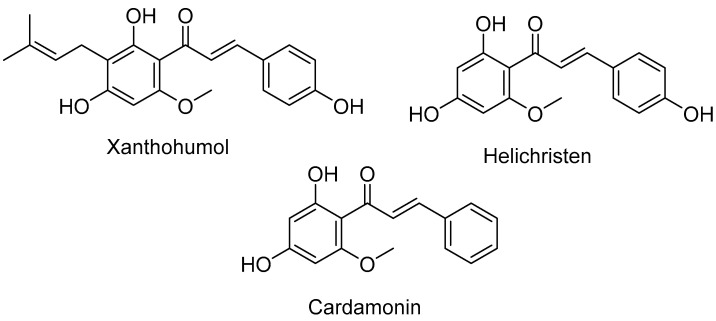
Structures of important anticancer chalcones.

Globally, cancer is a leading cause of death and disease, with over 10 million deaths annually [20]. To make the situation worse, there is a projected 47% increase in the incidence of cancer disease over the next 20 years [31]. Cancer is an often life-threatening condition that is caused by various mechanisms (immune, oxidative, genetic, or inflammatory) [32]. Despite the multitude of research studies conducted to cure various types of cancer, research cannot keep up with the constant mutation and changes in cell lines [32]. Additionally, since treatment has the goal of stopping division or eradicating malignant cells, there is a high number of detrimental side effects associated with traditional therapies like radiation or chemotherapy [33]. Classical chemotherapy often entails severe toxic side effects mainly because the selectivity of malignant cells is very low [31]. Furthermore, the increasing resistance against these therapies increases the need to discover new targeted treatment methods to reduce toxicity and increase the efficiency of eradication [20]. Due to their unique nature and bioavailability as small molecule inhibitors, natural chalcones have the potential to become new anticancer agents. Previous review articles have covered chalcones used for treating various types of cancers. One article focused solely on gastrointestinal (GI) cancer found that natural chalcones decrease enzyme activity, increase apoptosis, increase cell cycle arrest, increase reactive oxygen stress, decrease multidrug-resistant proteins, angiogenesis, and inflammation in gastrointestinal cancer [34]. However, this review focuses on carcinomas of the lungs, liver, and breast. This is partly because the three chalcones described are in the process of evaluating possible therapeutic agents for cancer as opposed to more traditional and potentially harmful options [20]. These natural chalcones (Figure 3) interfere with cancer processes through many mechanisms of action similar to the ones described for GI cancer [34]. Chalcones showed promising activity against breast cancer, liver cancer, lung cancer, and a multitude of carcinoma-related diseases. This section will focus on the three cancers listed and their remedial chalconoid molecules. 

### 5.1. Chalcones Being Investigated as Potential Anti-Breast Cancer Agents

Mainly affecting women, breast cancer is a complex heterogeneous disease with high incidence and mortality rates. Breast cancer features two types of tumor heterogenicity: inter-tumor heterogenicity and intratumor heterogenicity [35]. Thus, it is challenging to differentiate, including evaluations of the initial tumor, lymph nodes, metastasis, and individual cellular markers [31]. These markers are often immunohistological markers such as the estrogen and progesterone receptors (ER and PR), human epidermal growth factor receptor 2 (HER2), and others. Breast cancer is indicated explicitly by genomic markers such as breast cancer susceptibility proteins (BRCA1 and BRCA2) [35]. Among the various types of cancer, triple-negative breast cancer (TNBC) and inflammatory breast cancer exhibit the worst patient prognosis and treatment options [20]. Chalcones have shown activity that suppresses these malignancies, including suppression of breast cancer cell growth, adhesion, migration, and ability to metastasize [35]. For this reason, they are of key interest in developing new anticancer agents.

Xanthohumol (XH) is a chalcone from the hops plant (*Humulus lupulus*) and has numerous biological activities. Aside from targeting tumors, it is especially efficient at inhibiting the division of breast cancer [20]. Studies show that XH decreased cell proliferation based on the dosage amount. Xanthohumol is also mediated by its effect on cellular mitochondrial species. This was shown through the measurement of ROS species [31]. Decreasing cell proliferation was also dose-dependent; high levels of XH increased ROS production while low doses decreased it [32]. Xanthohumol also works by inhibiting tumor suppressor protein prohibitin2 by binding to brefeldin A-inhibited guanine nucleotide exchange protein 3 (BIG3). The inhibition released prohibitin2 to bind directly to nuclear and cytoplasmic receptors, suppressing estrogen signaling pathways and discouraging further tumor growth [35]. Further studies of the effect of XH on doxorubicin-resistant cancer cells (MCF-7/ADR) showed that xanthohumol inhibited growth, brought about apoptosis, and led to cell cycle arrest based on the dosage of XH. This was done to establish the relationship between xanthohumol dosage and its radiosensitizing activity [36]. The results from this study showed that XH sensitized MCF-7/ADR cells to radiation treatment by inducing apoptosis. The effect was combined with a decreased expression of proteins inhibiting apoptosis, further increasing the effect of xanthohumol [36]. Based on these results, it was concluded that xanthohumol could be used to sensitize cancer cells before treatment with radiation or chemotherapy, with the potential to increase significantly the outcome of previously ineffective treatments [36].

### 5.2. Chalcones as Anti-Liver Cancer Agents

More than 35% of deaths caused by cancer are associated with cancers of the digestive system, including liver cancer [35]. Liver cancer can stem from various factors, including chronic hepatitis B or C infection, excessive alcohol consumption, non-alcoholic fatty liver disease, exposure to aflatoxins, and certain genetic conditions [37]. The prognosis of liver cancer heavily depends on the stage at which it is diagnosed. Early detection significantly improves the chances of successful treatment. However, liver cancer is often diagnosed at advanced stages when treatment options are limited. The prognosis worsens as the cancer progresses, with advanced cases typically having a poorer outlook. Treatment options may include surgery, chemotherapy, radiation therapy, targeted therapy, or liver transplant, depending on the stage and severity of the cancer. During 2020, over 900,000 cases of liver cancer were diagnosed, and around 830,000 patients lost their lives to this cancer. Furthermore, there is a projected 56.4 increase in liver cancer diagnoses expected by 2040 [37].

HCC (hepatocellular carcinoma, also known as hepatoma) and HepG2 cells have inhibited cell proliferation by exposure to cardamonin (CAR), a natural chalcone found in Alpina Katsumadai [31]. CAR has been found to induce both the intrinsic and extrinsic pathways of apoptosis at an IC_50_ of 17.1 µM [38]. These effects are closely related to the intracellular generation of radical oxidative species (ROS) and inhibition of the Nf-kB pathway. The administration of cardamonin orally was proven to decrease tumor growth significantly and showed low levels of proliferation proteins [38]. These proteins include PCNA and Ki-67, as well as antiapoptotic Bcl-2 proteins, and there is an increase in the proapoptotic Bax moieties [31]. Based on these associations, the anticancer effect of CAR may be mediated through the Nf-kB pathway [31].

### 5.3. Chalcones as Potential Anti-Lung Cancer Agents

Lung cancer is primarily caused by exposure to carcinogens, with smoking being the leading risk factor. Approximately 85% of lung cancer cases are attributed to smoking, either directly through active smoking or indirectly through exposure to secondhand smoke. Other significant causes include exposure to environmental pollutants such as radon, asbestos, and air pollution. Genetic predisposition and family history also play a role, albeit to a lesser extent [35]. According to the American Cancer Society, lung cancer remains the leading cause of cancer-related deaths globally, with an estimated 2.2 million new cases and 1.8 million deaths reported in 2020 alone. The prognosis for lung cancer is often poor, with the five-year survival rate ranging from 5% to 20%, depending on the stage at diagnosis. Early detection through screening programs, smoking cessation interventions, and advancements in treatment modalities such as targeted therapies and immunotherapy are crucial in improving outcomes for individuals diagnosed with lung cancer.

Helichrysetin is a lesser-known chalcone found in plants of the genus Alpina and the flower *Helichrysum odoratissimum* [39]. Like other chalcones, helichrysetin exhibits antiproliferative effects against different tumor lines [20]. In A549 cells, mitochondrial-induced apoptosis is associated with DNA fragmentation and cell cycle arrest in the S phase [40]. Further study is needed on the IC_50_ and implementation of this compound into medical use [41].

## 6. Chalcones as Antidiabetic Agents

Research has shown that chalcones can enhance insulin sensitivity by activating the peroxisome proliferator-activated receptor gamma (PPARγ), a nuclear receptor crucial for insulin action. Additionally, chalcones have been found to inhibit α-glucosidase and α-amylase enzymes, responsible for carbohydrate digestion, thereby reducing postprandial hyperglycemia. Studies have also highlighted the role of chalcones in stimulating glucose uptake through the translocation of glucose transporter 4 (GLUT4) to the cell membrane, facilitated by the activation of AMP-activated protein kinase (AMPK) signaling pathway [42]. Furthermore, chalcones possess antioxidant properties that counteract oxidative stress, a hallmark of diabetes-associated complications. A study by Uddin et al. (2023) demonstrated the antidiabetic potential of chalcone derivatives through their ability to mitigate insulin resistance and β-cell dysfunction in diabetic rats [43]. This comprehensive understanding of the molecular mechanisms underlying chalcones’ antidiabetic effects underscores their therapeutic potential in managing diabetes and its complications.

**Figure 4:**
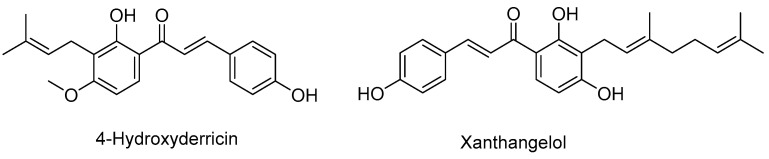
Two novel antidiabetic chalcones isolated from the plant *Angelica keiskei*.

In the Japanese herb “Ashitaba” *Angelica keiskei* (AE), over 20 medicinally active chalcones are present. Most chalcones are located in the root, stem, and leaf. Of the twenty chalcones identified in AE, two occur more frequently. These two chalcones are 4-hydroxyderricin and Xanthoangelol [44]. In the study conducted by Hu et al. (2018), the activity of the two chalcones found in AE in complimenting insulin action was examined. Upon examining the insulin-like activities of AE (including the 20 present chalcones), it was found that incubation of 3T3-L1 preadipocytes with AE resulted in the conversion of these preadipocytes to adipocytes in a dose-dependent manner [44]. It was also noted that differentiation into adipocytes did not occur without AE. Furthermore, AE did not exhibit toxicity even at high concentrations, as evaluated by the WST-1 assay. The two chalconoids, 4-hydroxyderricin (4-HA) and Xanthoangelol (XA) (Figure 4), were evaluated as potential causative agents in AE. These chalconoids were evaluated for inducing preadipocyte transformation and enhancing glucose uptake. In this test, the different chalcones were added to 3T3-L1 preadipocytes instead of insulin, and the effects were observed. The effect of 4-HD was almost equivalent to that of XA; however, the glucose uptake-enhancing activity of 4-HD was much higher than the uptake effect exhibited by XA [44]. These results indicate that the chalcones could induce preadipocyte differentiation to adipocytes and mimic insulin-like activities. Although these activities have been observed, the specific targets of 4-HD and XA have yet to be defined. They must have different targets, as 4-HD is more prominent in regulating glucose uptake than XA. Even at high doses, neither chalcone exhibited cytotoxicity, making the further study of their targets and mechanisms of action very enticing [44].

## 7. Anti-Inflammatory Chalcones

Chalcones possess notable anti-inflammatory properties. Among them, flavokavain A from the kava plant, xanthohumol from hops (*Humulus lupulus*) and the bark of certain trees, and isoliquiritigenin from licorice exhibit significant anti-inflammatory effects [45]. These compounds act through the inhibition of pro-inflammatory mediators such as nitric oxide (NO), prostaglandin E2 (PGE2), and cytokines like tumor necrosis factor-alpha (TNF-α) and interleukin-6 (IL-6). Moreover, they suppress the activation of key inflammatory signaling pathways, including nuclear factor kappa B (NF-κB) and mitogen-activated protein kinases (MAPKs) [46]. By targeting these pathways, chalcones mitigate inflammation, offering potential therapeutic benefits for conditions like arthritis and inflammatory bowel disease [47]. Their natural origin enhances their appeal for therapeutic development, though further research is necessary to fully understand their efficacy, safety, and clinical applications.

*Myracrodruon urundeuva* (*M. urundeuva*) or *Allemão anacardiaceae* is a medicinal plant used in northeast Brazil as a topical anti-inflammatory [48]. Three chalcones were isolated from the bark of *M. urundeuva* and tested together as a chalcone-enriched fraction (CEF). A study by Viana et al. (2003) created a report on the anti-inflammatory activity of these natural chalcones [48]. In a formalin test carried out in mice, the first phase (neurogenic) and the second phase (inflammatory), the CEF inhibited the second phase of the response. This is unsurprising because other studies have already demonstrated these effects with chalcones. Hsieh et al. (1998) emphasized the effect of chalcones on the release of histamine from rat mast cells when stimulated with the compound 48/80 [49]. They also reported that arachidonic acid-induced platelet aggregation was inhibited strongly while cyclooxygenase was inhibited, leading to a decrease in the production of prostaglandins [49]. It was concluded that because chalcones produce an anti-platelet effect, they result in the inhibition of thromboxane formation. *Myracrodruon urundeuva* and the CEF show similar functions but have not been studied in detail enough to know which cellular mechanism they target. The work completed by Viana et al. (2003) was the first known research into this specific CEF and showed promising results for future studies into the anti-inflammatory response of chalcones.

## 8. Chalcones as Neuroprotective Agents

Neurodegenerative diseases are diseases caused by various factors that ultimately lead to the progressive degeneration and death of neurons in the brain, spinal cord, and peripheral nervous system [50]. As these neurons are damaged or die, there is a loss of cognitive and motor function associated with neurodegenerative diseases [50]. Some common neurodegenerative diseases are Parkinson’s disease and Alzheimer's disease [51]. For example, one paper on Parkinson’s disease shows chalcones’ efficacy as enzyme inhibitors of MAO B and shows neuroinflammatory activity through Nrf2 activation. This is further emphasized by alternative pathways that can inhibit immune response and subsequent damage through iNOS inhibition and A1 and A2A receptor inhibition [52]. Monoamine oxidases are implicated in the degradation of amines that are necessary for emotional and cognitive brain function. A review of HMAO-B inhibitors showed that chalcones are efficient and regulation of molecular scaffolding can improve their performance by affecting different physiological and chemical features [53]. Although there are many treatments approved to manage the symptomology associated with neurodegenerative diseases, there are very few with significant real progress in curing or irradicating the issue [51]. Because of this, alternative treatments are more popular and generally going to be used. Multiple sclerosis (MS) is the most common autoimmune inflammatory neurodegenerative disease of the CNS and causes a loss of myelination of the axons. Several treatments exist for prevention and alleviating major symptoms; however, patients are ultimately still suffering due to the low efficiency of these treatments and their low therapeutic effects [51].

Chalcones have been investigated as promising agents for multiple sclerosis (MS) due to their neuroprotective effects [50]. The previously mentioned Japanese herb ashitaba has been shown to possess many secondary metabolites in chalcones with various therapeutic properties [54]. The small surface area and polarity of chalcones enable them to cross the blood–brain barrier (BBB), which further lends them to display pharmacological action in the central nervous system and an ability to protect local structures [55]. A study was conducted in which the chalcone isolated from ashitaba was incorporated into the diet of mice that had been given cuprizone to induce demyelination [50]. Upon investigation, the chalcone mixture notably lessened demyelination in the corpus colosseum and decreased TNFα levels in both the serum and brain of the chalcones from ashitaba-treated groups, compared to the group only given cuprizone [50]. Additionally, a higher dose of the chalcones from ashitaba markedly improved behavioral responses and boosted BDNF levels in the serum and brain of the ashitaba and cuprizone-treated group compared to the mice only treated with cuprizone [50].

Another chalcone used in treating neurodegenerative diseases is cardamonin [56]. Cardamonin (CD) is found in various plants, including *Alpinia gagnepainii*. As secondary metabolites, chalcones benefit their respective plants and are found in petals, leaves, and bark [56]. Because of its natural abundance, cardamonin (Figure 3) is not toxic upon consumption and can be used therapeutically without modification. The various therapeutic activities of CD lend it to further utilization as a neuroprotective agent against Alzheimer’s disease. Cardamonin has the potential to reduce microglia-derived oxidative stress [57]. Due to the origin of CD in plants from the *Zingiberacea* family, these leaves have been used as remedies for thousands of years [58]. These pharmaceutical properties limit the expression of iNOS and COX-2, giving rise to their anti-inflammatory properties. Furthermore, cardamonin inhibits the release of inflammatory cytokines, mainly TNF alpha [59]. The therapeutic effects of cardamonin have partly explained this by studying the NF-kB pathway in controlling DNA transcription and the immune response to infection [60]. Nrf2 activation, which is caused by cardamonin, supports the potential of CD as a possible therapeutic option for preventing neurodegenerative disorders caused by oxidative stress. Since most approved therapies target only a single gene or pathway, their effectiveness is limited due to the multifaceted nature of chronic diseases, which often involve disruptions in multiple cellular components and biological processes.

## 9. Cardioprotective Chalcones

In the United States, cardiovascular diseases (CVDs) are among the leading causes of morbidity. Since 1975, 1 in 4 deaths in America can be attributed to a type of CVD [61]. Additionally, CVDs are the leading global cause of death, with approximately 17.7 million deaths annually, according to the World Health Organization. Despite improvements in diagnosis and treatment reducing the age-adjusted rate and acute mortality from myocardial infarction, the risk of heart disease remains high, with a 50% risk by age 45. Incidence increases with age and is higher in younger men, but this gender difference narrows after menopause [61]. The drastic global increase in CVD can be associated with the large availability of fast foods, bad diet habits, and an overall reduction in exercise. In the case of cardiovascular diseases, prevention is better than treatment, and many cardioprotective compounds are found in foods associated with healthy diets. Many protective moieties are chalcones, such as quercetin, curcumin (a *bis*-chalcone derivative), and resveratrol. Such molecules give protection against myocardial infarction (MI) and other cardiovascular-related diseases [62]. Ailments like hyperlipidemia, hypertension, arrhythmia, and thromboembolism are offset by the strong antioxidant effect as well as inhibitory activities against lipid peroxidation and stimulation of cardioprotective protein production [63].

Because many chalcones occur naturally, they are low-cost and effective in preventing CVDs without much modification after extraction. For example, Ogawa et al. extracted an anti-hyperlipidemic chalcone from the plant *Angelica keiskei.* This chalcone, 4-hydroxyderricin (4-HD), was evaluated for its potential against lipid metabolism in stroke-prone hypertensive murine models [64]. It was reported that 4-HD improved the levels of HDL and caused a reduction in liver triglyceride content in the murine models. 4-HD also decreased liver weight as well as triglyceride levels after seven weeks of administration. There was also a large decrease in MTP, which may be associated with decreased VLDL levels [64]. All of this conclusively points to the potential of 4-hydroxyderricin in reducing hepatic triglyceride and elevated VLDL levels in stroke-prone hypertensive murine models [63]. Further investigation pinpointed the chalcone Xanthoangelol as a compound capable of decreasing LDL levels and the total cholesterol content. This may be due to an increase in fecal cholesterol excretion as well as the decreased liver weight. These effects may be brought about by the significant increase in PPAR-alpha mRNA expression, which is connected to an increase in the expression of acyl-coenzyme A synthetase and acyl-CoA oxidase mRNA expression [65].

Numerous natural chalcones interact with cardiovascular targets. For example, 2-hydroxychalcone, found in licorice root, and neobavachalcone, found in X, have potential benefits for ischemia-reperfusion injuries, vascular lesion regression, vascular injury response, and the prevention of coronary atherosclerosis. Other compounds, such as Licochalcone B or isoliquiritigenin, can lower hypertension and prevent MI and strokes through their beta-adrenergic receptor blockade action [66]. Similar activity has been demonstrated by glypallichalcone and licochalcone A in mixed receptor blockade activity through both alpha- and beta-adrenergic receptors. Somewhat of an anomaly, licochalcone G reports anti-thrombotic potential, which inhibits coagulation factor Xa. In summary, their adaptable structure and multifaceted unitality make natural chalcones vital in preventing the development of cardiovascular illnesses. They help prevent myocardial infarction, hyperlipidemia, hypertension, and other cardiovascular conditions through antioxidant and lipid-lowering effects.

## 10. Antimalarial Chalcones

Over half of the population of the world suffers from malaria. According to the WHO’s 2023 World Malaria Report, the estimated number of global malaria cases in 2022 exceeded pre-COVID-19 pandemic levels in 2019. In 2022, there were approximately 249 million malaria cases in 85 endemic countries, with an incidence rate of 58 per 1000 population at risk, compared to 233 million cases and an incidence rate of 57 per 1000 in 2019 [67]. This number is 55% higher than the target set by the 2025 Global Technical Strategy for Malaria, which aimed for an incidence of only 26 cases per 1000 population at risk in 2022. Globally, malaria caused an estimated 608,000 deaths in 2022, with a mortality rate of 14.3 deaths per 100,000 population at risk [67]. In particular, malaria, caused by the parasite *P. falciparum*, causes the most deaths and is prevalent in the region of Africa. For many years, chloroquine (CQ) was the treatment of choice for malaria. Its affordability to minimize host toxicity, paired with affordability, made it the standard therapy. Unfortunately, extensive use of CQ led to the emergence of CQ-resistant *P. falciparum* only a decade after the establishment of chloroquinone treatment [68]. Due to the spread of these resistant parasite strains, artemisinin-based combination therapy is the current treatment for *P. falciparum* strains [69]. However, the limited availability and development of resistance to artemisinin derivatives has made it apparent that new drugs are needed.

Since high rates of malaria are closely associated with poverty rates, novel antimalarial drugs that are both low-cost and effective are primarily sought after. Chalcones are part of an important family of secondary metabolites in plants and exhibit selective inhibition against *P. Falciparum*. This activity was first recorded for Licochalcone A [70]. Licochalcone A was isolated from licorice roots and, upon testing in murine models, was found to protect effectively against *P. Falciparum* infections when given orally or intraperitoneally for 3–6 days [70]. This compound was further investigated, and the mechanism was found to be related to the alteration of the parasite mitochondria, inhibiting their function [71]. Another study by Ziegler et al. showed the potency of licochalcone A as a membrane-active agent capable of transforming normal erythrocytes to echinocytes, which occurs with anti-plasmodial activity in a concentration-dependent manner [72]. This is key in protecting against malaria because it creates unfavorable conditions for the in vitro proliferation of the parasite. Crotaorixin is a chalcone isolated from parts of the plant *Crotalaria orixensis*. It exhibits 100% inhibition of maturation of the *P. falciparum* strain from the ring stage to the schizonts stage at concentrations of 50 and 10 µg/mL [73]. This chalcone is also being investigated for further modification into a pharmaceutical. Another well-known natural chalcone, xanthohumol, exhibited activity that interfered with hemin degradation in *P. falciparum*. This activity was shown at IC_50_ values of 8.2 to 0.3 (poW) and 24.0 to 0.8 mM (dD2) [73].

The search for novel antimalarial agents is critical to addressing the global challenge of malaria, particularly with the rise of drug-resistant strains. Natural chalcones are invaluable in creating new alternatives to combat resistant strains of malaria. As more and more drugs fall victim to the adaptation of malarial strains, more drugs and combinations are needed. The simple structure of chalcones makes them perfect for modification and staying ahead of a constantly mutating virus [74]. Compounds like licochalcone A, crotaorixin, and xanthohumol have shown significant potential in inhibiting the growth of *Plasmodium Falciparum* by targeting different stages of the parasite’s life cycle and interfering with crucial biological processes. The simplicity of chalcone structures facilitates their modification, enabling the development of more effective and affordable antimalarial drugs. As society continues to face the evolving threat of malaria, especially in regions with high poverty rates, investing in the research and development of chalcone-based therapies is essential.

## 11. Antibacterial Chalcones

Bacterial infection is an enormous threat to public health and one of the leading causes of death globally. These infections cause about 13.7 million fatalities, with 7.7 million caused by common bacteria [75]. Certain strains, namely *Staphylococcus aureus*, *Escherichia coli*, *Streptococcus pneumonia*, *Klebsiella pneumonia*, and *Pseudomonas aeruginosa,* are responsible for about 55% of these mortalities. Antibiotics are the primary treatment for bacterial infections and have a highly effective therapeutic action that can not only control the spread of but also entirely eradicate pathogenic bacteria [75]. Unfortunately, misuse and overuse of antibiotics have contributed to the emergence of antibiotic-resistant bacteria. This led to an arms war between the bacterium and novel antibiotics, ultimately resulting in a stalemate. Public health organizations are currently unable to meet the needs of the human population with existing antibacterial treatments [75]. In current research, many natural chalcones have been highlighted as potential antibacterial compounds; however, the known mechanisms of action have not yet been entirely uncovered [76].

In a review by Wang et al., various antibacterial natural chalcones and their mechanisms of action were discussed. They identified four mechanisms of action induced by natural chalcones and divided 17 natural chalcones into the corresponding categories [76]. Three aspects can be explored in the antibacterial activity of natural chalcones: antibacterial spectrum, antibacterial activity, and antibacterial mechanism [76]. The key finding of this review was the ability to identify which chalcones exhibit which antibacterial activity. Natural chalcones with antibacterial properties only against Gram-positive bacteria are 3-deoxypanchalcone, bavachalcone, echinatin, licochalcone B, licochalcone D, licochalcone E, pinocembrin chalcone and butien [76]. Other chalcones such as isobavachalcone, 4-hydroxylonchocarpin, licochalcone A, phloridzin, phloretin, and kanzonol C exhibit a broader antibacterial spectrum and can protect against Gram-positive bacteria, Gram-negative bacteria, and fungi [76]. Key examples will be discussed to better understand how chalcones evoke this antibacterial activity.

To sustain life, bacteria depend on membrane function and integrity. If the cell membrane is somehow damaged, bacterial growth will be halted and may lead to loss of life. Some of the normal life-sustaining processes present in bacteria require the assistance of proteins such as NADH oxidase, cytochrome c reductase, imidazole glycerol phosphate dehydratase (IGPT), efflux pumps, and countless other macromolecules [77]. The benefit of using natural chalcones to protect against bacterial infections is magnified by their ability to interfere with important macromolecules in metabolic pathways. For example, licochalcone A is a natural retro chalcone isolated from glycyrrhiza that exhibits the best antibacterial activity against Gram-positive bacteria with typical minimal inhibitory concentration MIC (minimum inhibitory concentration) values ranging from 1.56 to 12.5 µg/mL. Licochalcone hinders respiration in bacteria by inhibiting NADH oxidase combined with NADH-cytochrome c reductase activity [78]. Licochalcone A has also exhibited antibacterial activity against fungi with MIC values around 6.3–12.5 µg/mL [78]. The primary mechanism that licochalcone targets is the production of bacterial virulence factors. One study by Qiu et al. uncovered such a method of inhibition in *S. aureus* by inhibiting hemolysis by reducing the amount of alpha toxin produced [79]. While these findings highlight the potential of natural chalcones in combating bacterial infections, further research and clinical trials are necessary to fully exploit their antibacterial properties and develop them into effective therapeutic agents.

## 12. Antiviral Chalcones 

Viruses are biomechanical machines that reproduce using host cells. Recently, with the uproar of the COVID-19 pandemic, viruses have stepped into the academic spotlight again. Chalcones have been suggested as antivirals due to their overall resilience, as shown by their additional ability to inhibit viral reproduction. Chalcones possess the innate characteristic of stopping viruses in various portions of the replication cycle by inhibiting reverse transcriptase, proteases, neuraminidase, and aminotransferases [80]. The other reason that chalcones are studied as innovative antivirals is that they can be used when antiviral therapies are no longer viable due to the virus gaining resistance. Viral resistance occurs through genetic mutations that lead to phenotypic changes, leading to a previously effective drug losing efficacy [81]. This will then lead to higher mortality rates and increased infectivity.

Chalcones are pharmacologically active against a wide range of viruses, including coronavirus, influenza, human rhinovirus, dengue, herpes, HIV, cytomegalovirus, Rift Valley fever, hepatitis B and C, and Venezuelan equine encephalitis [80]. Influenza alone causes around half a million deaths worldwide annually [82]. Chalcones have been tested throughout major literature, and many different skeleton configurations are effective against various viral infections.

Severe acute respiratory syndrome coronavirus 2 is a highly infective virus responsible for the worldwide pandemic. Flavonoids are natural indole chalcones that have the pharmacokinetic property of inhibiting RNA-dependent polymerase, protease, and spike protein docking [83]. The types of flavonoids that have a specific inhibitory reaction to these major replicating proteins include cyanidin, which works on RNA polymerase, and Quercetin, which blocks the spike protein from interacting with the host cell [83]. Some studies suggest that the α, β-unsaturated ketone functional group allows chalcones to participate in Michael’s reaction and create strong covalent bonds to active proteins [84]. This means some chalcones can potentially create irreversible inhibition of the cysteine proteases and thus inhibit viral replication [84].

Chalcones can activate the NRF2 transcription factor, which is responsible for not only suppressing excessive inflammation through the suppression of inflammation but also regulating the antiviral responses of cells [85]. Isoliquiritigenin is a chalcone isolated from the roots and rhizomes of *Glycyrrhiza* spp., and it has been shown to have active suppression of HSV, hepatitis C, and influenza A [85]. Some chalcones, including curcumin, quercetin, and myricetin, have been seen to be noncompetitive inhibitors of the NS2b/NS3 protease in the dengue virus.

HIV has infected 70 million people worldwide and is still a leading cause of death in Africa [86]. Currently, antiretroviral therapies have been able to stop the progression of HIV but lead to severe side effects. Chalcones have been suggested as an alternative option. An example of this antiviral activity is the chalcone 2-Methoxy-3-methyl-4,6-dihydroxy-5-(3′-hydroxy) cinnamoyl-benzaldehyde which is from the *Desmos* spp., and it has been reported to inhibit HIV replication [87].

Overall, chalcones are versatile molecules with extreme pharmacokinetic abilities against viral agents. They bind to targets or defensive mechanisms of cells to help the body physiologically fend off the invasion. In addition to their unique chemical structure and attributes, chalcones are currently in the academic spotlight due to their prophylactic ability and extremely low toxicity [88].

## 13. Natural Chalcones in Clinical Trials

**Figure 5:**
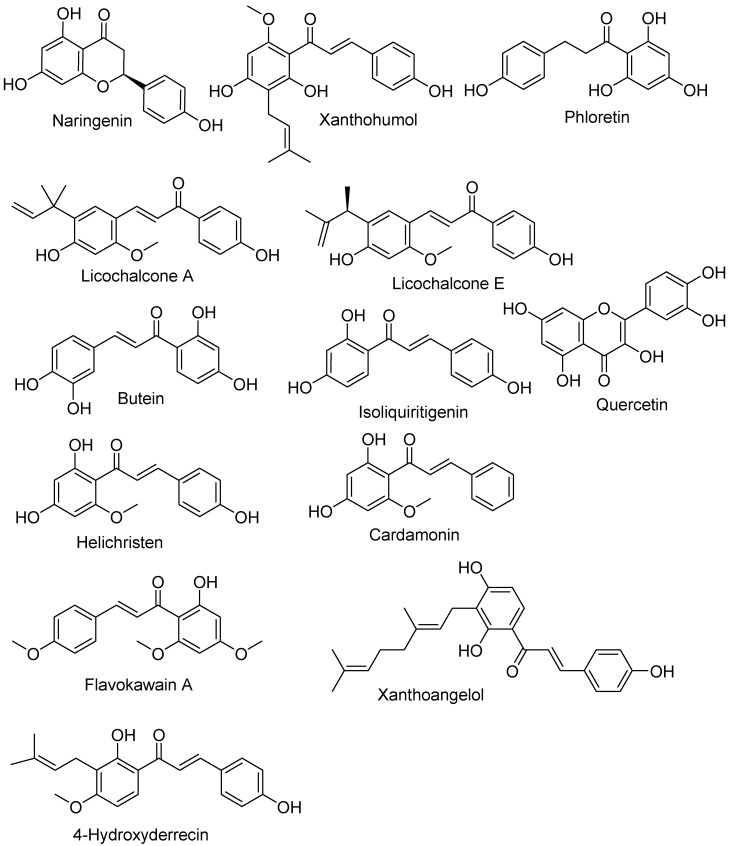
Representative examples of a few medicinally privileged natural chalcones.

Chalcones have many different properties that can be used in natural physiology. For example, in clinical trials using xanthohumol, which is found in the hop (*Humulus lupulus*), the availability in clinical trial is shown in Table 1 as well as structure of xanthohumol which is shown in Figure 5. 14 men were given either the chalcone or the placebo, and their peripheral blood mononuclear cells were isolated. The individuals who took xanthohumol induction of interleukin 1β, IL-6, and sCD14 protein through lipoteichoic acid showed similar results to unstimulated cells after 48 h. This effect is further seen at higher rates in HEK293-transfected cells [88]. Another clinical trial involving chalcone xanthohumol in healthy women also showed that it had different effects on the human body, such as reducing CD14 through the TLR4 signaling cascade, leading to a generalized anti-inflammatory effect [88].

Another way chalcones can be used is through effects on the physiological system, which include visceral fat deposits. In a study of 17 men and 25 women with a BMI greater than 25, participants took the chalcone ashitaba found in the plant *Angelica keskei*. After 8 to 12 weeks, visceral fat decreased more than the control groups, which was statistically significant. The clinical study concluded that the chalcone ashitaba did have anti-obesity effects on both men and women [89]. The chalcone flavokawain A from *Kaempferia angustifolia*, the Indonesian rhizome, a traditional weight-loss medicine, exhibited activity in reducing the differentiation of preadipocytes. This clinical study found that flavokawain C showed greater potency due to the electron-donating group on the B ring, which is suspected to be necessary for the molecule’s functionality [90]. A separate clinical study involving a variation of this chalcone found two effective chalcones, cardamonin and flavokawain B, which both inhibit the lipid accumulation in 3T3-L1 activation and modulate the differentiation of preadipocytes. Both effects are accomplished by ERK activation and the subsequent cascade that leads to the downregulation of transcription factors and adipokines [90]. This overall supports the effect of chalcones in decreasing obesity and the proliferation of adipocytes.

A single chalcone can also have multiple effects. For example, in a clinical study, 2,2′,5′-trihydroxy chalcone was selected for ROS scavenging in L-6 myoblasts and THP-1 human monocytes. It is usually used as an antioxidant, and this study showed that it has excellent antioxidant activity in a concentration lower than typically reported. This clinical study also showed the added benefit of inhibiting the proliferation of leukemic cells while not affecting myoblasts and human fibroblasts. Finally, the same study using Zucker fatty rats found that a 10 mg/kg dose of this chalcone led to decreased levels of glucose spikes and weight gain [91].

Chalcones also act as antiviral agents, such as the neuraminidase inhibitors from *Glycyrrhiza inflata*. A new licochalcone and seven known chalcones of the plant were tested on 293 T cells, and the compound in this study found that there were strong inhibitory effects on neuraminidases from influenza strains, including H1N1, H9N2, and antiviral resistant strains. These compounds can be used in the case of pandemics of oseltamivir-resistant strains of the H1N1 virus [91]. In another study, alkylated chalcones and coumarins were isolated from *Angelica keiskei* and were competitive inhibitors of SARS-CoV 3CL proteases [92]. Another study found that the same plant, *Angelica keiskei*, has three chalcones, including xanthokeistal A and 6,6-dimethoxy-3-methylhex-2-enyl that both inhibit influenza virus neuraminidase hydrolysis. The 2-hydroxy-3-methyl-3-butenyl alkyl (HMB) substituted chalcone was the most potent inhibitor, and all three were noncompetitive inhibitors [93]. A study on *Millettia leucantha* showed that dihydrochalcones derivatives from the stem bark show moderate anti-herpes simplex virus activity, while flavone 8 showed a significant anti-inflammatory effect on COX-1 and 2 [94].

**Table 1:** Important natural chalcones and their level of biological evaluations.

Natural Chalcones	Evaluation Phases, Including Clinical Trials				
	In Vitro	In Vivo	Phase I	Phase II	Phase III
Naringenin	[95]	[95]	[96]	n/a	n/a
Phloretin	[97]	[97]	[97]	n/a	n/a
Xanthohumol	[98]	[98]	[98]	[99]	n/a
Licochalcone A	[100]	[100]	[101]	[102]	n/a
Licochalcone E	[103]	[103]	n/a	n/a	n/a
Butein	[104]	[104]	n/a	n/a	n/a
Isoliquiritigenin	[105]	[105]	n/a	n/a	n/a
Quercetin	[106]	[106]	[107]	[108]	n/a
Helichristen	[40]	[40]	n/a	n/a	n/a
Cardamonin	[109]	[109]	n/a	n/a	n/a
Flavokawain A	[110]	[110]	n/a	n/a	n/a
Xanthoangelol	[111]	[111]	n/a	n/a	n/a
4-Hydroxyderrecin	[112]	[112]	n/a	n/a	n/a

## 14. Conclusions

This paper aims to comprehensively review natural chalcones, their pharmacological uses, and their roles in medicinal application. With their structurally diverse yet biologically active nature, chalcones offer various pharmacological properties, ranging from antioxidant to aphrodisiac effects. Our manuscript potentially deals with the pharmacologically privileged natural chalcones only. It does not include semi-synthetic chalcone analogs or synthetic compounds that contain chalcone scaffolds. In addition, it concisely discusses the most significant steps of the mechanism of action based on disease types. Understanding their biosynthetic pathways in plants, their classification, and mechanisms of action provides a solid foundation for understanding their therapeutic potential and further development in synthetic chemistry. Most chalcones are underutilized in their natural form and are instead obtained as synthetic derivatives. While this is useful, many potent health-beneficial chalcones that can be incorporated through diet already exist. Antioxidants such as xanthohumol and phloretin are present in foods, and consumption of these foods has been associated with better overall health. Furthermore, exploring natural chalcones’ roles in combating various diseases displays their importance in drug discovery and development. Specifically, their efficacy in targeting breast cancer, liver cancer, and lung cancer, as demonstrated by chalcones like xanthohumol and helichrysetin, suggests promising avenues for future therapeutic interventions. Additionally, the antidiabetic potential of chalcones, exemplified by studies on *Angelica keiskei*, gives hope for a more natural alternative for diabetes with lower toxicity. Future research on naturally occurring chalcones is likely to focus on the potential applications in pharmaceuticals in medicine and advancements in biotechnology. Scientists will explore natural chalcones and find ways to enhance them to provide better anti-inflammatory, antioxidant, and anticancer properties. Investigations and reviews of chalcones will find molecular mechanisms, pathways, and their effects on specific receptors to develop therapeutic efficiency. Additionally, research will continue to develop in the delivery of chalcones through different vectors to improve bioavailability. Computer modeling development will also improve the screening of chalcone libraries and accelerate the discovery and synthesis of specific chalcones with desired physio-chemical properties. All in all, different disciplines will drive research and allow natural chalcones to be further investigated and developed. The exploration of natural chalcones presented in this paper not only enhances understanding of their biological significance but also paves the way for developing novel therapeutics with improved efficacy and safety profiles.
